# Nutritional risk status and related influencing factors in patients with tuberculosis complicated with type 2 diabetes mellitus

**DOI:** 10.3389/fnut.2026.1691359

**Published:** 2026-02-11

**Authors:** Jing Cao, Hebin Xie, Zikai Yu, Sue Zhao

**Affiliations:** The Affiliated Changsha Central Hospital, Hengyang Medical School, University of South China, Changsha, China

**Keywords:** immune indicators, malnutrition, nutritional indicators, tuberculosis, type 2 diabetes mellitus

## Abstract

**Objective:**

To investigate the nutritional risk status of patients with tuberculosis complicated with type 2 diabetes mellitus (TB-T2DM) and analyze the related influencing factors.

**Methods:**

A retrospective study was conducted on adult patients (aged ≥18 years) with tuberculosis complicated with type 2 diabetes mellitus who were hospitalized in the Tuberculosis Department of Changsha Central Hospital from January to December 2024. A total of 196 patients were included in this study. The general demographic characteristics, nutritional indicators, immune indicators, clinical-related indicators of the research subjects, as well as the scores of Nutritional Risk Screening 2002 (NRS-2002) were collected. To analyze the nutritional risk status of patients with different types of tuberculosis, and a multivariate logistic regression model was used to analyze the influencing factors of nutritional risk.

**Results:**

Among the 196 patients, 46.43% were found to have nutritional risk. When categorized by tuberculosis diagnosis type, among the 179 patients with pulmonary tuberculosis combined with type 2 diabetes mellitus (PTB-T2DM), 48.60% had nutritional risk, while among the 17 patients with extrapulmonary tuberculosis combined with type 2 diabetes mellitus (EPTB-T2DM), 23.53% had nutritional risk. Multivariate logistic regression analysis showed that albumin, neutrophil to lymphocyte ratio, glycated hemoglobin, and body mass index (24.0–27.9 kg/m^2^) were independent influencing factors for TB-T2DM.

**Conclusion:**

Patients with TB-T2DM have a relatively high proportion of nutritional risk. Both nutritional and immune indicators have an impact on the occurrence of nutritional risk. In the clinical treatment process, more attention should be paid to the nutritional status of patients in order to reduce recurrence and improve prognosis.

## 1 Introduction

Tuberculosis (TB), caused by *Mycobacterium tuberculosis (M. tuberculosis)*, is one of the major infectious diseases worldwide. The Global Tuberculosis Report released by the World Health Organization (WHO) shows that 8.2 million new tuberculosis patients were registered and reported in 2023, reaching a historical high. China registered 564,900 patients, an increase of 12.7% compared with 2022, ranking third among countries with a high burden of tuberculosis (1). Relevant studies have shown (2) that factors such as malnutrition, smoking, poverty and diabetes are closely related to tuberculosis infection. Malnutrition, especially protein-energy malnutrition, can lead to abnormal metabolism and weakened functions of tissues and organs in the body, among which weakened immune function is particularly important (3). Cellular immunity is an important defense mechanism for the host against tuberculosis. Malnutrition leads to impaired cellular immunity, thereby increasing the body’s susceptibility (4, 5). On the other hand, *Mycobacterium tuberculosis* utilizes host proteins for its own metabolism. The bacteria and their metabolites cause consumptive changes such as repeated fever, night sweats, and weight loss in the body. All these lead to an increase in the basal metabolic rate of patients, a decrease in anabolism, and an increase in catabolism, thereby exacerbating malnutrition (6). To implement the “End TB Strategy,” the World Health Organization has proposed (7) that nutritional assessment and support for tuberculosis patients are indispensable in the diagnosis and treatment of tuberculosis.

Diabetes is a metabolic syndrome characterized mainly by hyperglycemia due to insufficient insulin secretion or dysfunction. Among them, type 2 diabetes mellitus (T2DM) accounts for approximately 95.0% of all diabetic patients (8). Recent studies have found that the incidence of tuberculosis combined with type 2 diabetes is 9.3–24.1%, showing an upward trend (9). These two diseases are closely related (10, 11). On the one hand, tuberculosis can promote metabolic disorders in diabetes, leading to unstable blood sugar levels and increasing the risk of complications; On the other hand, diabetes can lower the body’s immune function, increasing the risk of tuberculosis infection and the emergence of drug-resistant bacteria. Previous studies have found (12) that 20.7 to 42.50% of patients with tuberculosis complicated with diabetes suffer from malnutrition. In addition, studies have shown (13) that compared with patients without comorbidities, tuberculosis patients with diabetes have an increased risk of tuberculosis recurrence and nutritional risk, which seriously affects the prognosis of patients.

At present, there are few reports on the nutritional risk of patients with TB-T2DM. Therefore, this study aims to understand the nutritional status of patients with tuberculosis complicated with diabetes and analyze the related risk factors, providing a basis for identifying high-risk patients and conducting in-depth clinical nutritional therapy research in the future.

## 2 Methods

### 2.1 Inclusion and exclusion criteria

**Figure 1:**
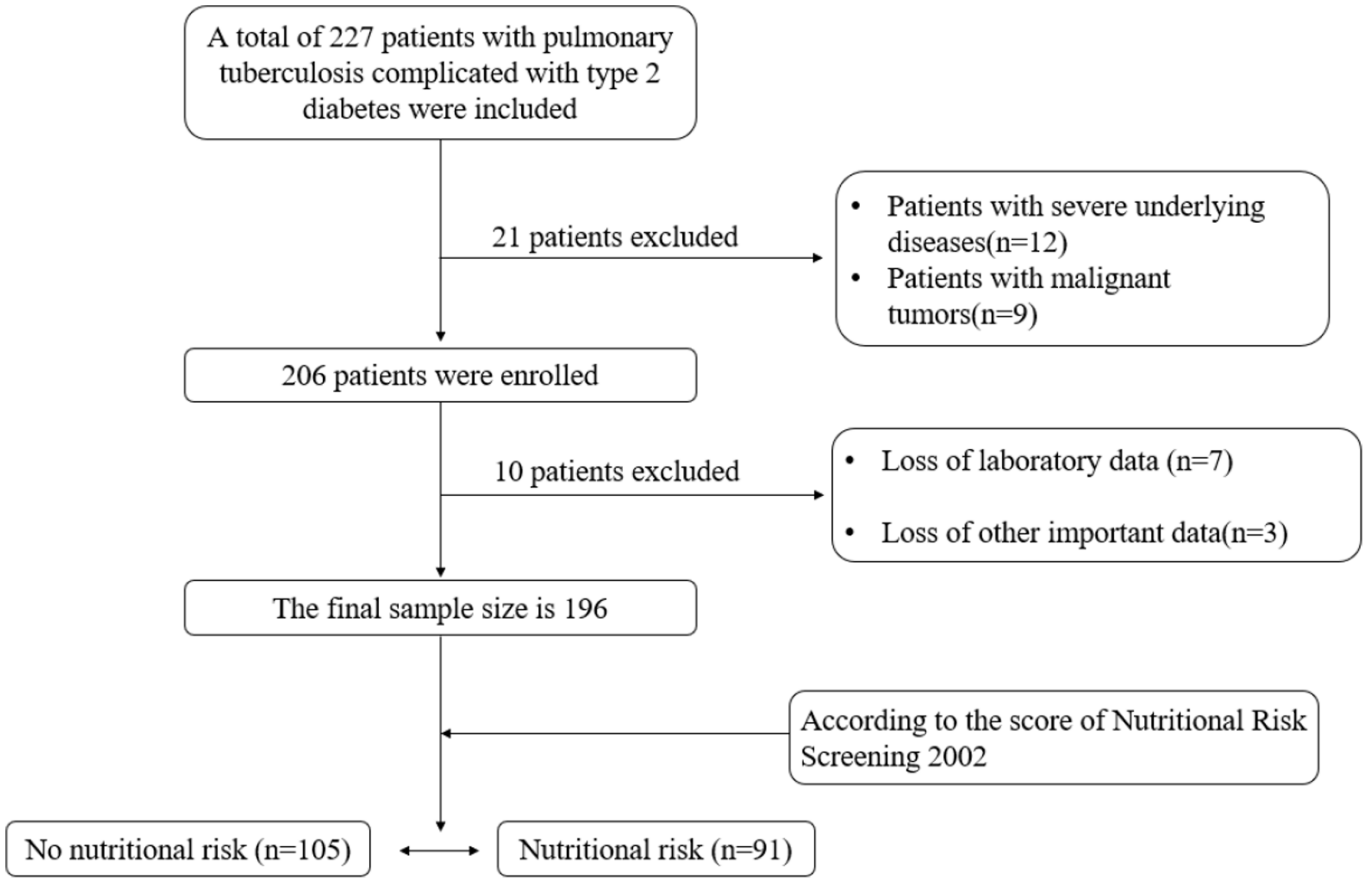
Flowchart of patient enrollment.

A retrospective study was conducted on adult patients (aged ≥18 years) with tuberculosis complicated with type 2 diabetes who were hospitalized in the Tuberculosis Department of Changsha Central Hospital from January to December 2024. A total of 196 patients were included (Figure 1). The diagnosis of tuberculosis conforms to the Chinese “WS 288–2017 Diagnosis of Pulmonary Tuberculosis” standard (14), the type of tuberculosis diagnosis refers to the Chinese “WS 196–2017 Classification of Tuberculosis” standard (15), and the diagnosis of type 2 diabetes is confirmed in accordance with the 2010 diabetes diagnostic criteria of the American Diabetes Association (16). Exclude patients with malignant tumors, mental disorders, hospital stays of less than 24 h, severe underlying diseases (such as chronic renal insufficiency, heart disease), and incomplete clinical data.

The study has been approved by the Human Ethics Review Committee of Changsha Central Hospital, and the study adhered to the principles of the Declaration of Helsinki (Ethical approval number:2021-S0177).

### 2.2 Research design and data collection

The nutritional risk of the patient was evaluated within 24 h after admission using the 2002 Nutritional Risk Screening (NRS-2002) (17). This tool consists of three separate scoring parts: ① impaired nutritional status (0–3 points), based on weight loss, BMI, and reduced dietary intake; ② disease severity (0–3 points), as our patients have active pulmonary tuberculosis, they receive a score of 1–3 points based on their specific conditions; ③ age adjustment (+1 point): 1 point will be added for patients aged ≥70. A total score of ≥3 points indicates that the patient has nutritional risk, while a total score of less than 3 points indicates no nutritional risk. Based on the NRS2002 scoring results, we divided the patients into the non-nutritional risk group (*n* = 115) and the nutritional risk group (*n* = 91).

Collect general patient information (including age, gender, educational level, and type of medical insurance) through electronic medical records and nursing systems. Nutritional indicators [including body mass index (BMI), serum total protein (TSP), albumin (ALB), hemoglobin (HGB), fasting plasma glucose (FPG), glycated hemoglobin (HbA1c), mean corpuscular hemoglobin (MCH), mean corpuscular volume (MCV)], immune indicators [neutrophil to lymphocyte ratio (NLR), platelet (PLT)], and clinical data (including length of hospital stay, type of tuberculosis diagnosis, whether it is initial treatment, whether there are complications, and so on).

### 2.3 Statistical analysis

All data were statistically analyzed using SPSS 25.0 software and R 4.4.0 software. The normality of continuous variables was tested through the Shapiro Wilke test as well as graphical illustrations of histograms and Q-Q graphs. Normal distribution continuous data were expressed as mean ± standard deviation (SD), and Independent-samples t-test was used for comparison between groups. Non-normally distributed measurement data were expressed as median (p25,p75), and the Mann Whitney test (non-parametric distribution) was used for comparison between groups. Categorical data were expressed as *n* (%), and the differences between the two groups were analyzed using chi-square analysis /Fisher’s exact test. The 95% confidence interval (CI) of the proportion (rate) of binary categorical variables was calculated using the Clopper-Pearson exact method.

The variables with significant initial screening (*p* < 0.05) were included in the multivariate Logistic regression model to determine the independent influencing factors of nutritional risk. Before constructing the final model, the variance inflation factor (VIF) is used to diagnose multicollinearity among independent variables. A VIF > 5 is considered to have significant multicollinearity. For missing data, first assess the proportion of missing data. The missing rates of the main analysis variables were all below 5%, and mean interpolation (for normally distributed variables) or median interpolation (for skewed distributed variables) was adopted for processing. To test the robustness of the core conclusion, we conducted the following sensitivity analysis: ① tighten the inclusion criteria for variables (*p* value threshold <0.05);② exclude extreme outliers in continuous variables (defined as exceeding the mean by ±3 standard deviations);③ variable form, convert key continuous variables (such as NLR) into binary variables based on commonly used clinical cut-off points (such as NLR = 4) and then re-model them. The statistical significance level of the two-sided test was 0.05.

## 3 Results

### 3.1 Patient nutritional risk status according to different tuberculosis diagnostic types

Among the 196 included patients, 153 were male (78.06%) and 43 were female (21.94%). The average age was 60.47 ± 11.67; a total of 91 patients were at nutritional risk (46.43%). We classified tuberculosis diagnosis types into pulmonary tuberculosis and extrapulmonary tuberculosis for statistics (15). Among 179 patients with pulmonary tuberculosis complicated with type 2 diabetes, 48.60% (95%*CI*: 40.8–56.4) had nutritional risks. Among the 17 extrapulmonary tuberculosis patients with type 2 diabetes mellitus, the proportion of those with nutritional risk was 23.53% (95%*CI*: 6.7–49.0). As shown in Table 1.

**Table 1:** Patient nutritional risk status according to different tuberculosis diagnostic types.

Type	*n*	No nutritional risk (*n* = 105)		Nutritional risk (*n* = 91)	
		*n*	Proportion (%)	*n*	Proportion (%)
**Pulmonary tuberculosis**	**179** ^a^	**92**	**51.40** ^a^	**87** ^a^	**48.60** ^a^
Hematogenous disseminated tuberculosis	20	8	40.00	12	60.00
Infiltrative tuberculosis	97	60	61.86	37	38.14
Cavitary tuberculosis	61	23	37.70	38	62.30
Tuberculoma	1	1	100.00	0	0.00
**Extrapulmonary tuberculosis**	**17** ^a^	**13** ^a^	**76.47** ^a^	**4** ^a^	**23.53** ^a^
Genitourinary tuberculosis	1	1	100.00	0	0.00
Bone tuberculosis	1	1	100.00	0	0.00
Tuberculous pleurisy	10	9	90.00	1	10.00
Tuberculous meningitis	4	1	25.00	3	75.00
Laryngeal tuberculosis	1	1	100.00	0	0.00
**Total**	**196** ^a^	**105** ^a^	**53.57** ^a^	**91** ^a^	**46.43** ^a^

^a^Represents the total number (N) and the percentage (%) of cases for pulmonary tuberculosis, extrapulmonary tuberculosis, and all tuberculosis diseases (including pulmonary tuberculosis and extrapulmonary tuberculosis).

### 3.2 Univariate analysis of nutritional risk in patients with TB-T2DM

The results of univariate analysis showed that there were statistically significant differences (*p* < 0.05) in tuberculosis diagnosis type, BMI, ALB, HGB, NLR, HbA1c, and average length of hospital stay between the two groups of patients, as shown in Table 2.

**Table 2:** Univariate analysis of nutritional risk in patients with tuberculosis complicated with diabetes.

Variable	Type	No nutritional risk (*n* = 105)	Nutritional risk (*n* = 91)	*χ^2^/t/z*	*p*
Gender, *n* (%)	Male	81(77.14)	72(79.12)	0.111	0.739
	Female	24(22.86)	19(20.88)		
Insurance type, *n*(%)	Medical insurance for urban workers	25(23.81)	19(20.88)	-	0.891^#^
	Medical insurance for urban residents	29(27.62)	29(31.87)		
	New rural cooperative medical	49(46.67)	42(46.15)		
	Others	2(1.90)	1(1.10)		
Degree of education, *n*(%)	Primary school	33(31.43)	40(43.96)	-	0.137^#^
	Junior high school	53(50.48)	42(46.15)		
	High school	16(15.24)	6(6.59)		
	College degree or above	3(2.86)	3(3.30)		
Tuberculosis type, *n*(%)	Pulmonary tuberculosis	92(87.62)	87(95.6)	3.924	0.048
	Extrapulmonary tuberculosis	13(12.38)	4(4.40)		
*Mycobacterium tuberculosis*, *n*(%)	Negative	65(61.90)	46(50.55)	2.559	0.110
	Positive	40(38.10)	45(49.45)		
Initial treatment, *n*(%)	No	82(78.10)	67(73.63)	0.534	0.465
	Yes	23(21.90)	24(26.37)		
Hypertension, *n*(%)	No	82(78.10)	66(72.53)	0.817	0.366
	Yes	23(21.90)	25(27.47)		
Other chronic lung diseases, *n*(%)	No	84(80.00)	71(78.02)	0.115	0.734
	Yes	21(20.00)	20(21.98)		
BMI, *n*(%)	<18.5 kg/m^2^	8(7.62)	16(17.58)	19.690	<0.001
	18.5–23.9 kg/m^2^	67(63.81)	70(76.92)		
	24.0–27.9 kg/m^2^	30(28.57)	5(5.50)		
Age, year (mean ± SD)		59.05 ± 11.61	62.11 ± 11.60	−1.843	0.067
MCH, pg. (mean ± SD)		30.15 ± 9.85	27.73 ± 8.04	1.864	0.064
MCV, fL (mean ± SD)		98.46 ± 18.09	93.86 ± 15.48	1.899	0.059
TSP, g/L, median (p25,p75)		63.80(59.20,69.70)	64.40(58.80,70.70)	−0.330	0.742
ALB, g/L,(mean±SD)		38.11 ± 3.51	33.06 ± 6.57	6.831	<0.001
HGB,g/L, median (p25, p75)		125.00(106.00,142.50)	115.00(105.00,125.00)	−2.677	0.007
NLR, median (p25,p75)		3.21(2.51,4.09)	4.24(2.87,5.94)	−4.039	<0.001
PLT,*10^9^/L, median (p25, p75)		262.00(241.50,291.50)	268.00(216.00,294.00)	−0.442	0.659
FPG, mmol/L, median (p25, p75)		6.95(6.15,8.21)	7.61(6.14,8.87)	−1.875	0.061
HbA1c,%, median (p25,p75)		7.38(5.81,8.99)	8.06(6.34,10.20)	−2.602	0.010
Average length of hospital stay, days, median (p25,p75)		8.00(7.00,9.00)	9.00(8.00,11.00)	−4.444	<0.001

^#^The Fisher’s exact probability method is adopted. Mean corpuscular hemoglobin (MCH), mean corpuscular volume (MCV), serum total protein (TSP), albumin (ALB), hemoglobin (HGB), fasting plasma glucose (FPG), glycated hemoglobin (HbA1c), neutrophil to lymphocyte ratio (NLR), platelet (PLT), body mass index (BMI).

### 3.3 Multivariate logistic regression analysis of nutritional risk in patients with TB-T2DM

The variables with statistically significant differences in the univariate analysis were included in the multivariate Logistic regression analysis model. The results showed that Albumin [*OR* = 0.825 (95%*CI*: 0.765–0.890)] and Glycated Hemoglobin [*OR* = 1.287 (95%*CI*: 1.081–1.534)] Neutrophil to lymphocyte ratio [*OR* = 1.535 (95%*CI*: 1.204–1.956)], BMI (24.0–27.9 kg/m^2^) [*OR* = 0.075 (95%*CI*: 0.016–0.357)] is an independent influencing factor for nutritional risk in patients with TB-T2DM (Table 3).

**Table 3:** Multivariate logistic regression analysis of nutritional risk in patients with TB-T2DM.

Variables	β	S. E.	Wald χ^2^	*p*	OR	95% C. I. for OR	
						Lower	Upper
Albumin	−0.192	0.039	24.503	<0.001	0.825	0.765	0.890
Hemoglobin	−0.003	0.009	0.124	0.725	0.997	0.980	1.014
Glycated hemoglobin	0.253	0.089	8.001	0.005	1.287	1.081	1.534
Average length of hospital stay	0.002	0.046	0.003	0.957	1.003	0.916	1.097
Neutrophil to lymphocyte ratio	0.429	0.124	12.000	0.001	1.535	1.204	1.956
Tuberculosis type (pulmonary tuberculosis)					1.000		
Tuberculosis type (extrapulmonary tuberculosis)	−1.314	0.809	2.641	0.104	0.269	0.055	1.311
BMI (<18.5 kg/m^2^)					1.000		
BMI (18.5–23.9 kg/m^2^)	−0.553	0.584	0.897	0.344	0.575	0.183	1.806
BMI (24.0–27.9 kg/m^2^)	−2.588	0.794	10.614	0.001	0.075	0.016	0.357

## 4 Discussion

TB-T2DM increases nutritional risk through synergistic pathophysiological interactions. Metabolically, insulin resistance and systemic inflammation cooperatively accelerate proteolysis, leading to muscle wasting (18, 19); immunologically, persistent immune activation depletes essential micronutrients, compromising nutritional status; and energetically, the hypermetabolic demand of TB combined with diabetic dysfunction creates a marked energy deficit, often exacerbated by reduced intake. Studies have found that these multi-faceted interactions may be based on the mammalian rapamycin target (mTOR) pathway, which is a central regulator of cell growth and metabolism in response to mitosis and nutritional signals (20, 21). Fan et al. (22) discovered that Rab1A is a key component in the amino acid sensing and signal transduction of the mTOR complex 1 (mTORC1), revealing the role and potential mechanism of amino acids in controlling the body’s glucose level through the *β* -cell-specific function of the RAB1A-mTORC1-PDX1 signaling axis. Chronic inflammation and insulin resistance in patients with TB-T2DM may inhibit the activity of mTOR complex 1 (mTORC1), thereby impening protein synthesis and exacerbating protein hydrolysis associated with muscle loss. The results of this study show that the prevalence of nutritional risk among patients with tuberculosis complicated with type 2 diabetes is 46.43%, which is consistent with previous reports (12). In addition, this study observed that patients with pulmonary tuberculosis exhibited a higher nutritional risk than those with extrapulmonary tuberculosis. Compared with patients with extrapulmonary tuberculosis (EPTB), the higher nutritional risk observed in patients with pulmonary tuberculosis (PTB) may be attributed to, on the one hand, the pulmonary symptoms (fever, cough) of PTB directly increase energy expenditure and hinder eating, leading to more severe metabolic expenditure and dietary difficulties. On the other hand, the potential underestimation caused by the limited sample size and internal heterogeneity within the EPTB group in this study.

ALB is a structural protein mainly synthesized by the liver, which can maintain the normal transmission of nutrients in the blood. ALB is a sensitive indicator for evaluating chronic protein deficiency. Relevant research reports (23) indicate that hypoproteinemia is an independent risk factor affecting the mortality rate of tuberculosis patients. When the body is in an inflammatory state and malnourished, the level of ALB will decrease, leading to a poor prognosis (24). This is consistent with the results of this study. The higher the ALB level, the lower the risk of nutritional occurrence. Therefore, nutritional intervention for individuals with low ALB levels before anti-tuberculosis treatment is a key factor influencing the condition and prognosis of tuberculosis.

This study shows that HbA1c and NLR are risk factors for malnutrition in patients. HbA1c represents the long-term level of blood glucose control, and a long-term hyperglycemic state leads to continuous oxidative stress and inflammatory responses in the body. Significant negative nitrogen balance in patients with type 2 diabetes is often associated with H-HbA1c levels (25). This imbalance in protein metabolism is caused by both an increase in protein degradation and clearance and a decrease in protein synthesis. Therefore, it leads to an increased risk of malnutrition in patients and further deterioration of insulin resistance, affecting prognosis. NLR is an important indicator for evaluating systemic inflammation and immune status. Multiple studies have reported (26–28) that NLR is an independent prognostic factor for lung cancer, breast cancer and gastric cancer. Stephenson et al. (29)’s study of 1,190 hospitalized elderly patients demonstrated that NLR is closely related to nutritional status, and this relationship can be attributed to inflammation as a direct catalyst for catabolic and nutrient distribution disorders. At present, there are relatively few reports on the nutritional risk status of patients with pulmonary tuberculosis and the changes in peripheral blood immune cell levels. This study initially explored that NLR is an independent risk factor for nutritional risk in patients with TB-T2DM, suggesting that the pro-inflammatory state indicated by high NLR may represent greater disruption of the patient’s immune balance, excessive consumption of energy and carbohydrate substances in the body, and an increased risk of malnutrition (30).

BMI is one of the most commonly used and intuitive indicators for nutritional risk screening and assessment in clinical practice. This study indicates that with the increase of BMI value, the incidence of nutritional risk in patients with TB-T2DM decreases. A retrospective study in South Korea (31) showed that the BMI of PTB smear-positive patients was lower than that of smear-negative patients. Badawi et al. (32) research has found that a high BMI index is negatively correlated with tuberculosis mortality, which means that obese people have a lower risk of dying from tuberculosis than those with normal weight or who are underweight. At present, this negative correlation mechanism is not fully understood. Nutritional status and adipose tissue may affect the body’s ability to resist tuberculosis infection by regulating immune system function (33). The role of cytokine-mediated innate immunity in the host’s resistance to *M. tuberculosis* infection has been confirmed in many infection experimental models, determining the key roles of interferon -*γ* (IFN-γ), tumor necrosis factor -*α* (TNF-α), and interleukin (IL) in controlling infection (34, 35). In addition, the research also found (33, 36) that malnutrition and subsequent acute or chronic weight loss are also considered factors influencing the development of tuberculosis. Nutritional status is closely related to the clinical prognosis of tuberculosis patients. Malnutrition not only affects the effectiveness and integrity of anti-tuberculosis treatment, but also significantly influences the quality of life and treatment costs of patients.

This study has certain limitations. Firstly, as a retrospective single-center study, we were unable to routinely collect the levels of micronutrients such as zinc and iron from patients, and also lacked prospective evaluations of dietary intake, body composition (such as muscle mass), or other objective nutritional biomarkers. This limited in-depth exploration of the specific mechanisms and types of malnutrition. Meanwhile, the single-center design and the limited overall sample size (*n* = 196) may affect the extrapolation of research conclusions to a broader population. Secondly, heterogeneity within the patient population may have an impact on the outcomes. The type 2 diabetes patients included in this study showed differences in disease duration, blood glucose control levels and hypoglycemic treatment regimens. This clinical heterogeneity may constitute unmeasured confounding factors. Furthermore, in the subgroup analysis, the number of patients with EPTB-T2DM was relatively small, especially for specific subtypes such as “tuberculoma” and “laryngeal tuberculosis,” there was only one patient each. This sample size was insufficient to support the results of the subgroup analysis. Therefore, the main conclusion of this study is more robustably applicable to patients with PTB-T2DM. Finally, the retrospective observational design was unable to infer causal relationships, nor could it assess the longitudinal association between nutritional status and long-term clinical outcomes, such as treatment success rate and survival rate. Therefore, in the future, it is necessary to conduct prospective, multi-center, and large-sample studies to systematically collect multi-dimensional nutritional indicators and detailed clinical metabolic data, and to accumulate rare subgroups over the long term, in order to verify this finding and deeply clarify its causal mechanism.

## 5 Conclusion

The results of this study reveal that 46.43% of patients with TB-T2DM have nutritional risks. Pulmonary tuberculosis patients with nutritional risk [48.60% (95% *CI*: 40.8 5–6.4)] is higher than the extrapulmonary tuberculosis [23.53% (95% *CI*: 6.7–49.0)]. ALB and BMI (24.0–27.9 kg/m^2^) are protective factors for malnutrition in patients with TB-T2DM, while glycated hemoglobin and NLR are risk factors for malnutrition in patients with TB-T2DM.

## Data Availability

The raw data supporting the conclusions of this article will be made available by the authors, without undue reservation.
